# Arterial wall fibrosis in Takayasu arteritis and its potential for therapeutic modulation

**DOI:** 10.3389/fimmu.2023.1174249

**Published:** 2023-05-15

**Authors:** Durga Prasanna Misra, Kritika Singh, Aman Sharma, Vikas Agarwal

**Affiliations:** ^1^ Department of Clinical Immunology and Rheumatology, Sanjay Gandhi Postgraduate Institute of Medical Sciences (SGPGIMS), Lucknow, India; ^2^ Clinical Immunology and Rheumatology Services, Department of Internal Medicine, Post Graduate Institute of Medical Education and Research (PGIMER), Chandigarh, India

**Keywords:** Takayasu arteritis, giant cell arteritis, fibrosis, T lymphocyte, fibroblast, mechanistic target of rapamycin complex 1

## Abstract

Arterial wall damage in Takayasu arteritis (TAK) can progress despite immunosuppressive therapy. Vascular fibrosis is more prominent in TAK than in giant cell arteritis (GCA). The inflamed arterial wall in TAK is infiltrated by M1 macrophages [which secrete interleukin-6 (IL-6)], which transition to M2 macrophages once the inflammation settles. M2 macrophages secrete transforming growth factor beta (TGF-β) and glycoprotein non-metastatic melanoma protein B (GPNMB), both of which can activate fibroblasts in the arterial wall adventitia. Mast cells in the arterial wall of TAK also activate resting adventitial fibroblasts. Th17 lymphocytes play a role in both TAK and GCA. Sub-populations of Th17 lymphocytes, Th17.1 lymphocytes [which secrete interferon gamma (IFN-γ) in addition to interleukin-17 (IL-17)] and programmed cell death 1 (PD1)-expressing Th17 (which secrete TGF-β), have been described in TAK but not in GCA. IL-6 and IL-17 also drive fibroblast activation in the arterial wall. The Th17 and Th1 lymphocytes in TAK demonstrate an activation of mammalian target organ of rapamycin 1 (mTORC1) driven by Notch-1 upregulation. A recent study reported that the enhanced liver fibrosis score (derived from serum hyaluronic acid, tissue inhibitor of metalloproteinase 1, and pro-collagen III amino-terminal pro-peptide) had a moderate-to-strong correlation with clinically assessed and angiographically assessed vascular damage. *In vitro* experiments suggest the potential to target arterial wall fibrosis in TAK with leflunomide, tofacitinib, baricitinib, or mTORC1 inhibitors. Since arterial wall inflammation is followed by fibrosis, a strategy of combining immunosuppressive agents with drugs that have an antifibrotic effect merits exploration in future clinical trials of TAK.

## Introduction

Tissue injury resulting from transmural arterial inflammation in Takayasu arteritis (TAK) is consequent to the infiltration of different immune cells, including macrophages, dendritic cells, neutrophils, mast cells, and T lymphocytes. Such arterial wall inflammation heals with fibrosis, resulting in stenosed arteries with a distorted microarchitecture (1–3). For reasons not yet clear, vascular inflammation in TAK heals with a greater degree of fibrosis (resulting in arterial stenosis) than in the counterpart large vessel vasculitis (LVV) of giant cell arteritis (GCA), where arterial dilatation occurs more often (4–6). Stenosis in TAK can result in critical downstream ischemia (7) leading to myocardial infarction or ischemic stroke (7) which can occur even during inactive disease (8).

Relapses frequently occur when corticosteroids are tapered in TAK (9). Therefore, maintenance immunosuppressive therapy with disease-modifying anti-rheumatic drugs (DMARDs), whether conventional, biologic, or targeted synthetic, is usually initiated along with corticosteroids (10–14). However, quite unlike GCA, no immunosuppressive therapy has been proven to be beneficial against a placebo in a randomized controlled trial of TAK to date (13–15). To date, there are no validated clinical measures of damage (which may reflect vascular fibrosis) in TAK (15). However, angiographic scoring systems are available for TAK, which reflect vascular damage well (16). Angiographic extent of TAK might increase over time despite having inactive disease clinically (17). This necessitates lateral thinking to explore newer therapeutic avenues for TAK to target both arterial wall inflammation and vascular remodeling (18). Given the prominence of arterial wall fibrosis following the resolution of inflammation in TAK (2, 4–6), we review the literature regarding the pathogenic mechanisms driving arterial fibrosis in TAK and their potential for therapeutic modulation.

## Cellular populations contributing towards arterial fibrosis in TAK

### Macrophages

**Figure 1:**
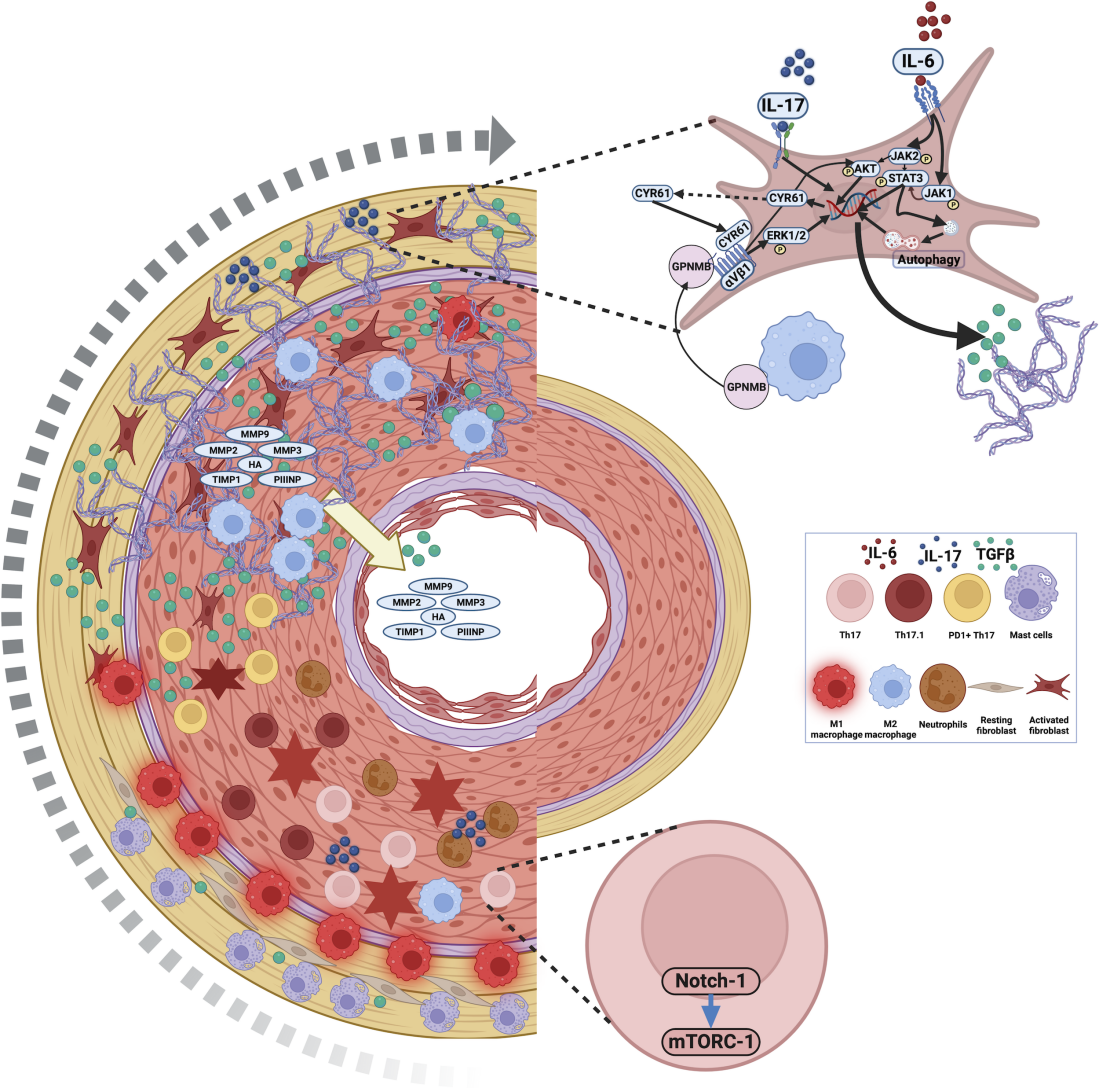
Immune processes resulting in vascular fibrosis in Takayasu arteritis. Mast cells, M1 macrophages, and T lymphocytes infiltrate the arterial wall in TAK. Activation of Notch-1 with downstream activation of the mammalian target organ of rapamycin complex 1 (mTORC1) is a key mechanism driving the activation of Th1 and Th17 lymphocytes in the inflamed arterial wall of TAK. Mast cells, PD1+ Th17 lymphocytes (which secrete TGF-β), macrophages (source of IL-6), and Th17 and Th17.1 lymphocytes (through IL-17 secretion) activate the resting fibroblast population. Th17 lymphocytes also recruit neutrophils to the inflamed arterial wall resulting in tissue destruction. The activated fibroblast population secretes greater amounts of extracellular matrix and also releases further TGF-β into the inflamed arterial wall. Over time, the inflammatory M1 macrophage population transitions to an M2 phenotype that further secretes TGF-β. M2 macrophages also secrete glycoprotein non-metastatic melanoma protein B (GPNMB), which also drives fibroblast activation. Tissue injury and fibrosis in the arterial wall may be reflected by circulating levels of TGF-β; hyaluronic acid (HA); matrix metalloproteinases (MMP) 2, 3, and 9; tissue inhibitor of metalloproteinase 1 (TIMP-1); and pro-collagen III amino-terminal pro-peptide (PIIINP). Created with BioRender.com.

Infiltration of inflammatory M1 and reparative M2 macrophages (19) into the inflamed arterial wall is recognized in TAK. Kong et al. identified a preponderance of M1 macrophages and localization of the macrophage chemoattractant C-C motif ligand 2 (CCL2) in the adventitia of the arterial wall of TAK in immunosuppressive-naïve TAK. After immunosuppression, M2 macrophages were more prevalent in the media of the arterial wall where CCL-2 was now majorly expressed (as opposed to the adventitial layer previously) (20). Similarly, Cui et al. also reported a dominance of M1 macrophages coexistent with arterial wall inflammation and M2 macrophages coexistent with arterial wall fibrosis in TAK. The addition of leflunomide (but not corticosteroids) to peripheral blood monocytes of TAK cultured *in vitro* with monocyte colony-stimulating factor (M-CSF) reduced M2 macrophage polarization relative to M1 macrophages. Methotrexate only at higher doses inhibited M2 polarization to a similar extent as leflunomide. Cultured M2 macrophages in the presence of leflunomide secreted lesser CCL22 and transforming growth factor beta (TGF-β) into the culture supernatant. Leflunomide also induced M2 macrophage apoptosis. The same authors further used the THP-1 monocyte cell line polarized towards M2 macrophages using IL-4 and IL-13. Upon treatment with leflunomide, the cultured macrophages showed a reduced expression of *IL10* and *IRF4* genes (associated with M2 polarization). Pro-fibrotic gene expression (*TGFB1*, *PDGFB*, and *LAGLS3*) was also reduced with leflunomide, mediated by decreased STAT6 phosphorylation (21). Thus, leflunomide reduced M2 macrophage polarization and inhibited their pro-fibrotic phenotype *in vitro* (21). The role of macrophages in the vascular fibrosis of TAK is summarized in **Figure 1**.

### Mast cells

Mast cells are involved in tissue repair (22). Le Joncour et al. demonstrated elevated circulating markers of mast cell activation in TAK compared to healthy controls. Furthermore, they stimulated *in vitro* cultured mast cells with interleukin-33 (IL-33) with or without sera of TAK or healthy controls and treated cultured arterial wall fibroblasts with the supernatant of stimulated mast cells. Platelet-derived growth factor (PDGF) and TGF-β were elevated in the culture supernatant of mast cells incubated with TAK sera than those incubated with sera from healthy controls, unaffected by IL-33 inhibition or IL-6 inhibition. Cultured arterial wall fibroblasts treated with mast cell culture supernatant incubated with TAK sera had increased expression of markers of fibroblast activation, i.e., collagen 1, fibronectin, and alpha-smooth muscle actin (α-SMA) when compared with those treated with sera from TAK alone. Furthermore, greater activation of cultured arterial fibroblasts was observed with mast cell culture supernatant incubated with TAK sera than from healthy controls. These findings suggested a role for mast cells in driving the pathogenesis of vascular wall fibrosis in TAK (23). The role of mast cells in the vascular fibrosis of TAK is summarized in **Figure 1**.

### T lymphocytes

Mammalian target organ of rapamycin complex 1 (mTORC1) is a therapeutic target for fibrosis (24–26). Zhang et al. reported an increased frequency of Th1 and Th17 lymphocytes when naïve CD4+ T lymphocytes from TAK were cultured *in vitro* when compared with those from healthy controls or with granulomatosis with polyangiitis (GPA, a small vessel vasculitis). Cultured CD4+ T lymphocytes from TAK demonstrated mTORC1 activation when compared with healthy controls or with GPA. *In vitro* treatment of CD4+ T lymphocytes from TAK with the mTORC1 inhibitor rapamycin or silencing of mTORC1 RNA expression reduced the frequencies of Th1 and Th17 lymphocytes. Circulating CD4+ T lymphocytes expressing mTORC1 moderately correlated with acute phase reactants erythrocyte sedimentation rate (ESR) and C-reactive protein (CRP). In a murine model of TAK using human axillary arteries implanted into NSG mice infused with peripheral blood mononuclear cells (PBMCs) from active TAK, treatment with rapamycin or silencing of mTORC1 RNA expression reduced arterial wall inflammation *in vivo* (27). Jiang et al. further identified Notch-1 upregulation in CD4+ T lymphocytes from TAK compared to healthy controls or GPA. Inhibiting Notch-1 expression using the gamma-secretase inhibitor DAPT or silencing RNA reduced Th1 and Th17 lymphocyte differentiation in cultured CD4+ T lymphocytes from TAK. Furthermore, Notch-1 mediated Th1 and Th17 lymphocyte polarization *via* mTORC1 activation (28). Maciejewski-Duval et al. further reported mTORC1 activation (identified by phosphorylation of S6 ribosomal protein) in the adventitia of LVV arteries (more in TAK than in GCA), which co-localized with CD3+ and CD4+ lymphocyte infiltration. Greater mTORC1 activation was evident in Th1 than in Th17 lymphocytes from TAK, GCA, and healthy controls. Th17 lymphocytes with mTORC1 activation were more prevalent in TAK than in GCA. Upon culturing PBMCs from TAK *in vitro* with rapamycin, decreased frequencies of Th1, Th17, and IL-21-expressing CD4+ T lymphocytes were observed than without rapamycin (29).

Programmed cell death 1 (PD1) was previously thought to be a marker of T lymphocyte exhaustion (30). However, a seminal report implicated PD1+CD4+ T lymphocytes and PD1+Th17 lymphocytes as key drivers of fibrosis *via* TGF-β1 secretion in idiopathic pulmonary fibrosis and sarcoidosis-associated pulmonary fibrosis (31). Elevated Th17 lymphocytes are associated with disease activity in TAK (32–34). Follicular helper T lymphocytes (TFH) that also express PD1 (35) are elevated in TAK (36). Tertiary lymphoid organs (TLOs) were more often identified in the arteries of TAK than of GCA. Increased PD1 expression was also noted in TLOs from the arterial wall of TAK when compared with TLOs from arteries affected by GCA (36). Work from our group identified elevated CD4+ PD1+ T lymphocytes in the peripheral blood of TAK than in healthy controls or sarcoidosis and increased PD1+ Th17 lymphocytes in TAK than in healthy controls. CD4+ PD1+ T lymphocytes and PD1+ Th17 lymphocytes did not significantly differ before or after immunosuppressive therapy in TAK (34). IL-23 helps to maintain the differentiated Th17 cell population (37). Interestingly, a previous genome-wide association study of TAK implicated the *A* allele in the rs6871626 single-nucleotide polymorphism (SNP) in the *IL12B* region as a risk allele for TAK (38). Furthermore, those TAK who were homozygous or heterozygous for the A allele of rs6871626 had greater vascular damage as indicated by higher scores on the vasculitis damage index (VDI) and the TAK Damage Score (TADS) (39). These observations suggest that Th17 lymphocytes, particularly the PD1+ Th17 subset, might drive vascular fibrosis in TAK. Inhibiting mTORC1 activation on T lymphocytes through rapamycin, sirolimus, or everolimus might target vascular fibrosis in TAK (13, 40). The role of T lymphocytes in the vascular fibrosis of TAK is summarized in **Figure 1**.

## Aortic adventitial fibroblasts as drivers of fibrosis in TAK

IL-6 drives both vascular inflammation and vascular fibrosis in TAK. Kong et al. reported the co-localization of IL-6 and IL-6 receptor (IL-6R) with alpha-smooth muscle actin (α-SMA) in the arterial wall adventitia of TAK. Increased proliferation of human aortic adventitial fibroblasts (AAFs) was observed upon treatment *in vitro* with a combination of IL-6 and IL-6R, coupled with increased production of α-SMA, collagen 1, collagen 3, fibronectin, and TGF-β1, mediated through JAK2 acting downstream mainly through STAT3 and also *via* AKT (41). Chen et al. reported that IL-6 co-localized with Atg3 (a marker of autophagy) and α-SMA in the TAK aortic wall adventitia. Thereafter, they observed *in vitro* induction of autophagy in AAF upon treatment with IL-6 and IL-6R. The fibrotic phenotype of AAF induced by treatment with IL-6 and IL-6R could be reversed with the late-phase autophagy inhibitor bafilomycin A1, with decreased collagen-1 and fibronectin in the culture supernatant. Addition of JAK1 inhibitors tofacitinib and itacitinib to IL-6 and IL-6R *in vitro* reduced LC3-II expression (a marker of autophagy), autophagosome formation, and production of collagen 1 and fibronectin in the culture supernatant of AAF, mediated through STAT3 (42). These experiments identify potential mechanisms for the observed effectiveness of tocilizumab (12) and tofacitinib (11) in TAK.

Th17 lymphocytes, particularly the PD1+ Th17 population, had been associated with TAK (34). Ma et al. further dissected the potential role of IL-17 in the vascular fibrosis of TAK. They reported increased cysteine-rich protein 61 (CYR61) expression in the adventitia of arteries from TAK. Upon stimulating the AAF with CYR61, increased production of fibronectin, collagen 1, collagen 3, and TGF-β1 were observed, mediated by αVβ1 receptor *via* ERK1 and ERK2. Treatment of AAF with recombinant human IL-17 stimulated CYR61 secretion. Co-culture of AAF with IL-17 and CYR-61 markedly enhanced the pro-fibrotic phenotype when compared with CYR61 alone (43).

A GWAS study identified the *A* allele in the rs2069837 SNP in the *IL6* region as a risk allele for TAK (44). This SNP repressed glycoprotein non-metastatic melanoma protein B (GPNMB), which has anti-inflammatory effects (44, 45). Interestingly, M2 macrophages that are more prevalent in fibrotic arteries than in inflamed arteries of TAK secrete GPNMB (46). Dai et al. identified GPNMB co-expression with collagen 1, fibronectin, TGF-β, matrix metalloproteinase 2 (MMP2), and MMP9 in the adventitial layer of arteries from TAK than from non-inflammatory controls. In the TAK arterial wall, GPNMB co-localized with macrophages and fibroblasts. The culture supernatant of THP-1 macrophages overexpressing GPNMB induced the expression of collagen 1, fibronectin, TGF-β, MMP2, and MMP9 in AAF when compared with the culture supernatant from THP-1 macrophages with a knock-down of GPNMB. After treating AAF with soluble GPNMB in the culture media, a similar overexpression of collagen 1, fibronectin, TGF-β, MMP2, and MMP9 with increased fibroblast proliferation and migration was observed. Opposite effects were observed after knocking down GPNMB in the AAF. The effect of GPNMB on AAF was mediated *via* the αVβ1 receptor acting downstream on AKT and ERK-1/2 pathways. *In vitro* treatment of AAF with GPNMB along with leflunomide, tofacitinib, or baricitinib reduced collagen 1, fibronectin, TGF-β, MMP2, and MMP9 expression when compared with GPNMB alone. Leflunomide also suppressed GPNMB production from THP-1 macrophages *in vitro*. However, in 22 patients with TAK treated with leflunomide and corticosteroids, inconsistent changes in circulating GBNMB were observed (47). The role of adventitial fibroblasts in the vascular fibrosis of TAK is summarized in **Figure 1**.

## Circulating proteins as biomarkers of fibrosis in TAK

Kong et al. identified, among numerous serum chemokines, an increase in CCL22 (produced by M2 macrophages) (48) following immunosuppressive therapy, whereas the levels of IL-16 did not change following immunosuppressive therapy (49). CCL22 has been implicated previously in lung fibrosis (50) and IL-16 in cardiac fibrosis (51). The persistent elevation of these chemokines in TAK despite immunosuppressive therapy might also contribute towards vascular fibrosis while inflammation resolves.

Another recent study explored the enhanced liver fibrosis (ELF) score, a validated circulating biomarker of liver fibrosis derived from serum levels of hyaluronic acid (HA), tissue inhibitor of metalloproteinase 1 (TIMP-1), and pro-collagen III amino-terminal pro-peptide (PIIINP), in 24 patients with TAK. The ELF score moderately correlated with clinical damage indices VDI and TADS and strongly correlated with angiographically assessed vascular damage using the Combined Arteritis Damage Score (52).

MMP2, MMP3, and MMP9 are secreted by fibroblasts during tissue remodeling, including in the inflamed arterial wall. Some studies (15) but not others have associated circulating levels of MMP2, MMP3 (53), and MMP9 with TAK disease activity (54). Whether MMP2, MMP3, and MMP9 are associated with vascular fibrosis in TAK remains to be evaluated. **Supplementary Table S1** summarizes potential biomarkers for further evaluation of their association with vascular fibrosis in TAK.

## Differences in the pathogenesis of TAK and GCA—are differences in Th17 subtypes a key feature?

Vascular fibrosis is prominent in affected arteries of TAK but less so in GCA (2, 4–6). Th17 lymphocytes were initially implicated in GCA (55). Subsequent reports identified a role for Th17 lymphocytes in TAK (32–34), including an association with active TAK (32, 34). Th17 lymphocytes attract neutrophils to the arterial wall (56). Recently, IL-17 has been implicated in granuloma formation (57, 58), a pathological feature of both TAK and GCA (2). Th17.1 lymphocytes implicated in TAK (34) also secrete interferon-gamma (classically secreted by Th1 lymphocytes) (59), which also drives granuloma formation (60).

The Th17 population in GCA is corticosteroid-responsive (55). However, a reduction in Th17 lymphocytes following immunosuppressive therapy was not observed in TAK (32, 33). The poor responsiveness of Th17 lymphocytes in TAK could be explained by the elevated Th17.1 population known to express the drug efflux protein p-glycoprotein (thereby conferring corticosteroid resistance) (34, 61). Th17.1 lymphocytes have not yet been described in GCA (61). Despite elevated tumor necrosis factor-alpha (TNF-α) levels in both TAK (62) and GCA (63), TNF-α inhibitors (TNFi) are effective in TAK (10) but not in GCA (64). Blocking TNF-α inhibits p-glycoprotein expression (65), which might be one of the mechanisms driving the effectiveness of TNFi in TAK.

**Figure 2:**
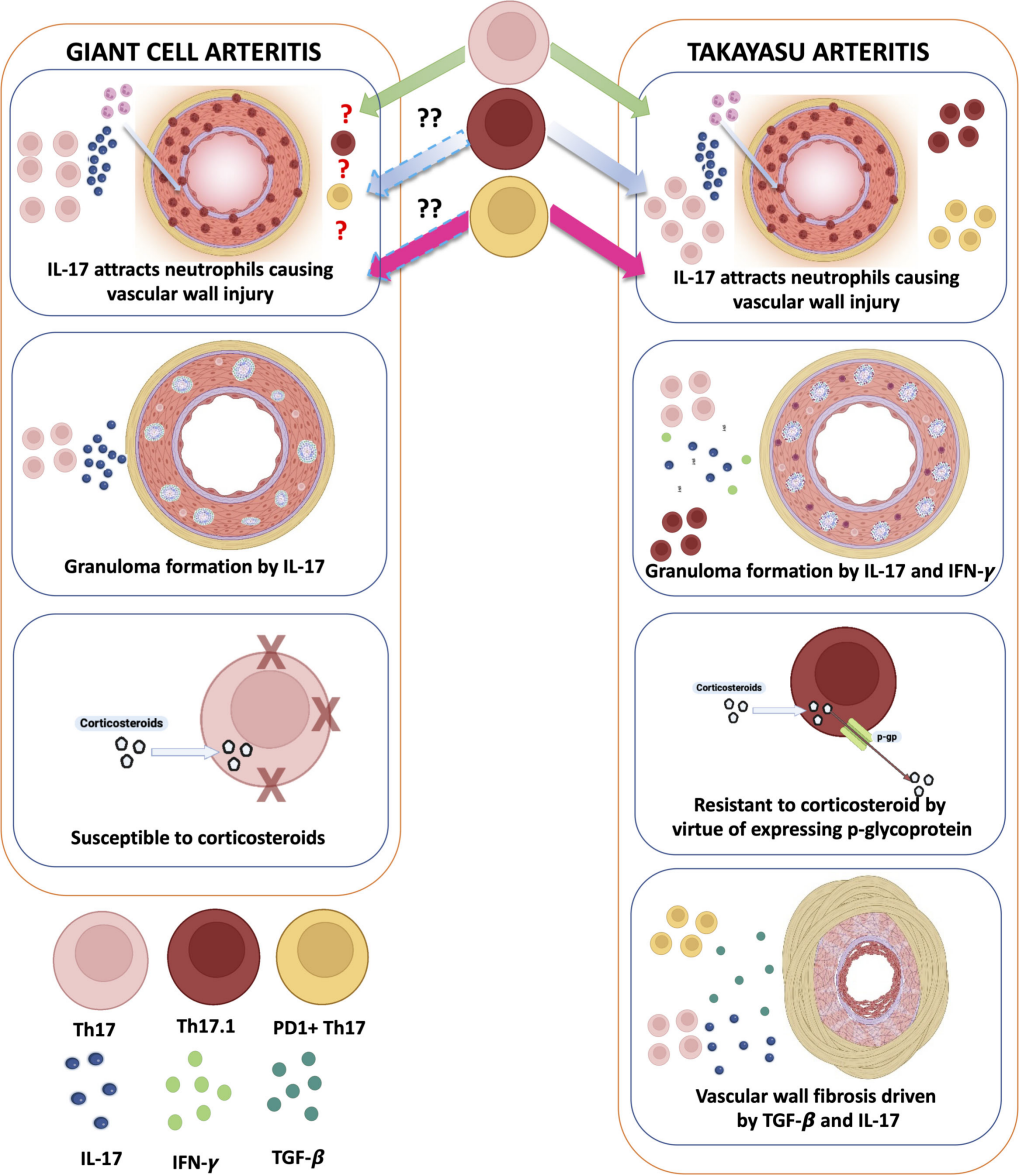
Distinct roles of Th17 lymphocytes in giant cell arteritis and Takayasu arteritis: do they underlie mechanistic differences in vascular fibrosis? In giant cell arteritis (GCA), Th17 lymphocytes, which are sensitive to corticosteroids, predominantly secrete IL-17, which attracts neutrophils causing inflammation of the vascular wall. IL-17 also contributes to granuloma formation in the vascular wall. In Takayasu arteritis (TAK), apart from these roles, Th17.1 lymphocytes also secrete IFN-γ (contributing towards granuloma formation) and express p-glycoprotein (contributing towards corticosteroid resistance). Th17 lymphocytes expressing programmed cell death 1 (PD1) also secrete TGF-β1, which, along with IL-17, contributes towards vascular fibrosis. Created with BioRender.com.

PD1+ Th17 lymphocytes secrete TGF-β, which drives arterial wall fibrosis in TAK (34) along with IL-17 secreted by Th17 lymphocytes. PD1+ Th17 lymphocytes have not yet been described in GCA. Such differences in Th17 sub-populations could explain distinct vascular pathology encountered in TAK or GCA (**Figure 2**).

## Detection of fibrosis *in vivo* in Takayasu arteritis

Computed tomography (CTA), magnetic resonance (MRA), or conventional angiography helps delineate the arterial tree anatomy in TAK and other LVV. CTA and MRA also provide information about arterial wall characteristics, more easily appreciated when intravenous contrast is administered during angiography (15). Late gadolinium enhancement of the arterial wall on MRA may indicate either inflammation or fibrosis (66, 67).

18-Fluorodeoxyglucose (^18^F-FDG) positron emission tomography (PET), combined with computed tomography (CT) or more recently magnetic resonance imaging (MRI) for anatomic localization, is increasingly being used to visualize metabolic activity in the arterial wall in TAK (15). A recent paper proposed that concomitant arterial wall ^18^F-FDG uptake and wall thickening evident on MRA might indicate ongoing inflammation, whereas wall thickening without ^18^F-FDG uptake might indicate vascular fibrosis (68).

68-Gadolinium (68-Ga)-tagged fibroblast activation protein inhibitor (FAPI)-PET identifies *in vivo* fibroblast activity in solid organ tumors or lungs in the context of interstitial lung diseases (69). A case report described a young female with clinically active TAK without 18-FDG-PET uptake but with extensive arterial uptake using 68-Ga-FAPI-PET (70). It remains to be explored whether 68-Ga-FAPI-PET can enable the identification of areas of ongoing arterial fibrosis in TAK.

## Prospects to therapeutically target arterial fibrosis in Takayasu arteritis

mTORC1 activation drives the differentiation to Th1, Th17, and IL-21+CD4+ T lymphocytes in TAK (27–29). Elevated PD1+ Th17 lymphocytes in TAK serve as a source of TGF-β (34). mTORC1 activation has also been noted in the endothelial cells of TAK but not in GCA. Purified immunoglobulin G1 (IgG1) from TAK induced endothelial cell proliferation *in vitro* through the PI3K/AKT pathway, inhibited by the mTORC1 inhibitor sirolimus or through a direct inhibitor of PI3K (71). Sirolimus and everolimus are mTORC1 inhibitors commonly used in clinical practice (40). mTORC1 inhibition has antifibrotic effects in kidneys affected with antiphospholipid antibody syndrome (25). mTORC1 inhibition has also been proposed as a therapeutic modality for pulmonary fibrosis (24). Therefore, mTORC1 inhibition might ameliorate both inflammation and fibrosis in TAK. Sirolimus has been anecdotally used in TAK (72). Leflunomide suppresses M2 macrophages and aortic adventitial fibroblasts *in vitro* (21, 47). The JAK inhibitors tofacitinib and baricitinib also suppressed aortic adventitial fibroblasts *in vitro* (47).

Sirolimus-coated stents inhibit endothelial proliferation and reduce re-stenosis after endovascular stenting. A case report described a patient with TAK with repeated coronary artery stenosis despite a sirolimus-eluting stent where the addition of systemic corticosteroids prevented restenosis from occurring with a sirolimus-eluting stent alone (73). This suggests the potential benefits of combining immunosuppressive therapy with antifibrotic drugs in TAK.

To explore this concept further, we treated PBMCs from TAK cultured *in vitro* with tacrolimus (a calcineurin inhibitor) and tadalafil (a phosphodiesterase 5 inhibitor that increases intracellular levels of cyclic guanosine monophosphate, thereby suppressing canonical and non-canonical signaling pathways downstream to TGF-β). Upon stimulation of the PBMCs with anti-CD3/CD28, treatment with tacrolimus and tadalafil significantly reduced the levels of IL-6, IL-17A, IL-1β, and IL-10 in the culture supernatant than tacrolimus alone (34). IL-6 (41, 42) and IL-17 (43) activate aortic adventitial fibroblasts in TAK. IL-10 (74) and IL-1β (75) have been associated with organ fibrosis in clinical and pre-clinical models. The synergistic effect of tacrolimus and tadalafil on various cytokines involved in fibrosis *in vitro* on cultured PBMCs from TAK (34) suggests the potential to explore a combination of immunosuppressive and antifibrotic therapies in TAK. Given that no clinical trial of an immunosuppressive agent in TAK has met its primary endpoint, future trials should consider combining DMARDs with antifibrotic drugs.

## Conclusion

Excessive vascular fibrosis characterizes TAK. Th17 lymphocyte populations, M2 macrophages, and mast cells drive aortic adventitial fibroblast activation leading to arterial wall fibrosis. *In vitro* experiments suggest the potential to target arterial wall fibrosis in TAK with leflunomide, tofacitinib, baricitinib, or mTORC1 inhibitors. Since arterial wall inflammation begets fibrosis, a strategy of combining immunosuppressive agents with antifibrotic drugs merits exploration in future clinical trials of TAK.

## Author contributions

Conception and design of the study: DM, AS, and VA. Acquisition of data, analysis, and interpretation of data: DM and KS. Drafting the article: DM and KS. Critically revising the article for important intellectual content: AS and VA. Final approval of the version to be submitted: DM, KS, AS, and VA. Agreement to be accountable for all aspects of the work in ensuring that questions related to the accuracy or integrity of any part of the work are appropriately investigated and resolved: DM, KS, AS, and VA. All authors contributed to the article and approved the submitted version.
