# Supplementary material for: Arterial wall fibrosis in Takayasu arteritis and its potential for therapeutic modulation
Source: Front Immunol. 2023 May 15;14:1174249. doi: 10.3389/fimmu.2023.1174249 (PMC10225504; doi:10.3389/fimmu.2023.1174249)
Supplement: Supplementary file 1 [file Table_1.docx]

**Supplementary Table S1: Potential biomarkers of vascular fibrosis in Takayasu arteritis**

| Cell populations | PD1+ CD4+ T lymphocytes |
| --- | --- |
|  | PD1+ Th17 lymphocytes |
| Circulating proteins | CCL-2 |
|  | CCL-22 |
|  | IL-16 |
|  | GBNMB |
|  | TGF-𝞫1 |
|  | PDGF |
|  | Hyaluronic acid |
|  | PIIINP |
|  | TIMP-1 |
|  | MMP-2 |
|  | MMP-3 |
|  | MMP-9 |
